# Incidence of Dementia Before Age 65 Years Among World Trade Center Attack Responders

**DOI:** 10.1001/jamanetworkopen.2024.16504

**Published:** 2024-06-12

**Authors:** Sean A. P. Clouston, Frank D. Mann, Jaymie Meliker, Pei-Fen Kuan, Roman Kotov, Lauren L. Richmond, Tesleem Babalola, Minos Kritikos, Yuan Yang, Melissa A. Carr, Benjamin J. Luft

**Affiliations:** 1 Program in Public Health, Stony Brook University, Stony Brook, New York; 2 Department of Family, Population, and Preventive Medicine, Renaissance School of Medicine at Stony Brook University, Stony Brook, New York; 3 Department of Applied Mathematics & Statistics, Stony Brook University, Stony Brook, New York; 4 Department of Psychiatry, Renaissance School of Medicine at Stony Brook University, Stony Brook, New York; 5 Department of Psychology, Stony Brook University, Stony Brook, New York; 6 World Trade Center Health Program, Commack, New York; 7 Department of Medicine, Renaissance School of Medicine at Stony Brook University, Stony Brook, New York

## Abstract

**Importance:**

Reports suggest that the individuals who served in rescue operations following the terrorist attacks on the World Trade Center (WTC) have poorer brain health than expected.

**Objective:**

To assess the incidence of dementia before age 65 years in a prospective study of WTC responders and to compare incidence among responders with severe exposures to debris vs responders not exposed to building debris or who wore personalized protective equipment (PPE).

**Design, Setting, and Participants:**

This prospective cohort study was conducted from November 1, 2014, to January 1, 2023, in an academic medical monitoring program available to verified WTC responders residing on Long Island, New York. Responders 60 years of age or younger without dementia at the time of their first cognitive assessment were followed up every 18 months, on average, for up to 5 years.

**Exposures:**

Exposure severity was based on responses to a detailed questionnaire of WTC exposures and exposure-related activities that included exposures to fine particulate dust and potentially neurotoxic debris, duration of work, and the use of PPE. Exposure level was divided into 5 categories ranging from low to severe.

**Main Outcomes and Measures:**

Incidence of all-cause dementia before age 65 years was the primary outcome. Dementia was diagnosed following standard guidelines relying on repeated measures of cognition.

**Results:**

Of 9891 responders, 5010 were eligible for inclusion in this study of cognitive function (median [IQR] age, 53 [48-57] years; 4573 [91.3%] male). There were 228 cases of dementia identified during 15 913.1 person-years of follow-up. Increasing WTC exposure severity was associated with incremental increases in the incidence rate of dementia per 1000 person-years (low, 2.95 [95% CI, 1.07-11.18]; mild, 12.16 [95% CI, 10.09-14.79]; moderate, 16.53 [95% CI, 13.30-20.81]; high, 30.09 [95% CI, 21.35-43.79]; and severe, 42.37 [95% CI, 24.86-78.24]). Adjusting for social, demographic, and relevant medical factors, each unit increase in exposure severity was associated with increased incidence of dementia (adjusted hazard ratio, 1.42 [95% CI, 1.18-1.71]; *P* < .001; mean risk difference, 9.74 [95% CI, 2.94-32.32] per 1000 person-years; *P* < .001).

**Conclusions and Relevance:**

In this cohort study of WTC responders who survived these unique exposures and participated in a longitudinal follow-up study of cognition from 2014 through 2022, when compared with responders with the lowest exposure levels or responders who used PPE, more severe exposure to dust or debris was significantly associated with a higher risk of dementia before 65 years of age. This study suggests that the reliable use of PPE might help prevent the onset of dementia before age 65 years among individuals exposed to an uncontrolled building collapse. Future research is warranted to determine cerebral biomarkers for individuals with exposure-associated dementia.

## Key Points

**Question:**

Is there an association between occupational exposures while responding to the World Trade Center disaster and the incidence of dementia before 65 years of age?

**Findings:**

In this cohort study including 5010 World Trade Center general responders aged a median of 53 years at initial assessment, the incidence of dementia before age 65 years was higher in responders who were more severely exposed. Compared with minimally exposed responders who reported no dust exposure or used personal protective equipment, responders working on the pile of debris who reported severe exposures to dust had a higher incidence of dementia before age 65 years even after adjusting for demographic, medical, and social factors.

**Meaning:**

Disasters often require an emergent response in dangerous conditions, but reliable use of PPE might help prevent the onset of dementia before age 65 years among individuals exposed to an uncontrolled building collapse.

## Teaser

This cohort study compares the incidence rate of dementia before 65 years of age among responders with severe exposure to debris with responders not exposed to building debris or who wore personalized protective equipment during rescue operations following the terrorist attacks on the World Trade Center.

## Introduction

The terrorist attacks on the World Trade Center (WTC) on September 11, 2001, caused the uncontrolled collapse of the iconic Twin Towers, which expelled dust, chemicals, and other detritus across lower Manhattan, New York.^1^ During response efforts, workers reported heavy exposure to dust and particulate matter that caused acute gastrointestinal and respiratory discomfort and decreased pulmonary functioning.^2^ Long-term exposure to inhaled air pollutants, including particulate matter, has been identified as a potential risk factor for the earlier onset of dementia,^3^ and has been proposed as a cause of dementia in WTC-affected populations.^4^ Consistent with this finding, an emerging body of research identified evidence of cognitive dysfunction,^5^ cognitive decline,^6^ and widespread cerebral atrophy among severely exposed WTC responders at midlife as compared with expectations.^7^ Changes in cognition and cerebral atrophy are established risk factors for dementia.^8^ However, the incidence of dementia before the age of 65 years in the general population is estimated to be fairly rare (incidence rate [IR], 1.19 per 1000 person-years).^9^ To date, the importance of variability in exposures during WTC response efforts has been interrogated with respect to cancer outcomes,^10^ but the role of exposures to dust and the use of personal protective equipment (PPE) in disaster clean-up work in incident dementia is unknown. To determine the incidence of dementia, we repeatedly assessed cognition and functional limitations in a cohort of WTC responders living on Long Island, New York, who were 60 years of age or younger when the first cognitive assessment was completed. We hypothesized that responders with more severe exposures to airborne particulate matter would have worse cognitive outcomes.

## Methods

The institutional review board Committee on Research Involving Human Participants at Stony Brook University, Stony Brook, New York, reviewed and approved of this cohort study. All procedures were completed following the protocol, and all responders provided informed written consent. This report follows the Strengthening the Reporting of Observational Studies in Epidemiology (STROBE) reporting guideline for cohort studies.^11^

### Setting

Individuals who worked or volunteered in lower Manhattan, the Staten Island landfill, or on barge loading piers for at least 4 hours in the period from September 11 through September 14, 2001, for 24 hours at any other time in September, or for at least 80 hours across the entire response period from September 11, 2001, through July 31, 2002,^12^ were considered WTC responders.^13^

We created the present nested cohort study to assess cognition among consenting WTC responders who attended monitoring appointments on Long Island, New York, starting November 1, 2014, with an average responder completing an initial cognitive assessment before April 31, 2016. Data collection waves occurred 18 months apart, on average, and the first follow-up assessments began on December 1, 2015. Since the parent cohort is open to enrollment, this study also continued enrolling responders into cognitive assessments until January 1, 2020, to allow sufficient follow-up time for diagnosis prior to January 1, 2023, after accounting for clinic shutdowns in 2020 and 2021. A power analysis (eMethods in Supplement 1) suggested that at least 5000 responders with 5 years of follow-up could facilitate studying incidence of dementia before age 65 years.

### Inclusion and Exclusion Criteria

**Figure 1.**
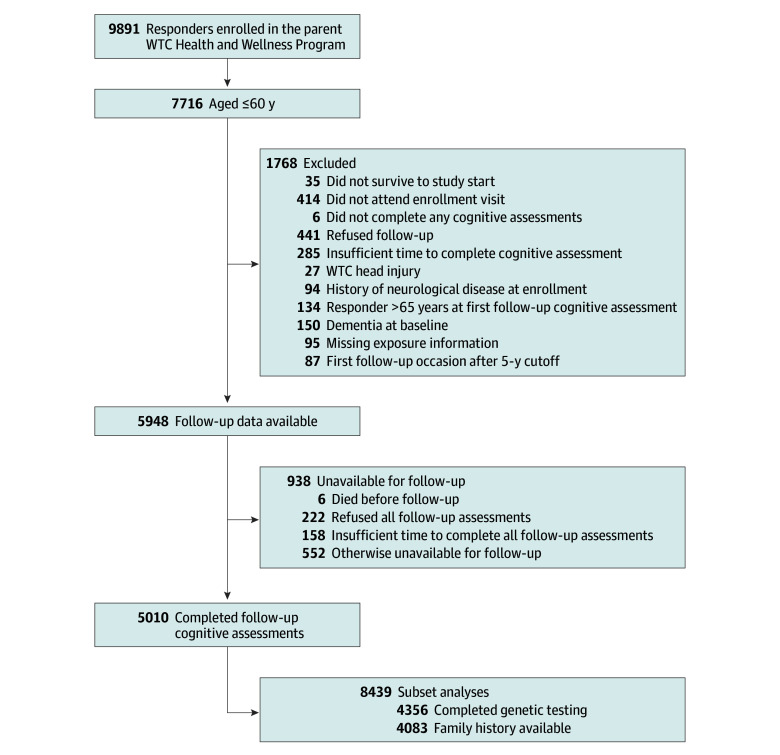
Recruitment Flowchart for the World Trade Center (WTC) Responder Cohort

Briefly, responders who were aged 60 years or younger at baseline, fluent in English (>99% of responders listed English as their primary language) and survived to attend at least 2 to 5 monitoring visits from November 1, 2014, to January 1, 2023, were eligible for inclusion (Figure 1). Eligible participants were excluded from the analysis if they had experienced a head injury during operations at the WTC, had a prevalent neurological or cerebrovascular diagnosis, including a diagnosis of all-cause dementia, at their initial cognitive assessment, or if they refused all follow-up cognitive assessments.

### WTC Exposure Severity

Prior work in this field has predominantly used the duration of WTC exposure as a proxy for exposure severity,^14^ although 1 study of WTC tower survivors who did not work in rescue or response activities also identified associations between WTC dust exposure and cognitive functioning.^15^ The parent responder monitoring program began enrolling patients July 1, 2002, as soon as possible after exposure ended. At study intake, responders were asked to fill out a detailed exposure assessment questionnaire that assessed the type of activities that responders engaged in while working at the WTC site, their location of work, the duration of work, and the types of exposures they experienced while working.

Exposure assessments commenced July 1, 2002, and were completed, on average, by December 31, 2010. Recognizing that prior work has suggested that timing and duration of WTC exposures could modify levels of exposure^4^ and that levels of exposure might also be made more severe by working in more dangerous response activities, we began by defining a low probable exposure category to include responders whose exposures were least likely to include physical interaction with potentially neurotoxic dust, such as those who were working in clean rooms, and those who routinely wore PPE, including hooded Tyvek or Hazmat suits and respirators or masks.

To identify higher exposure activities, we took 2 overall approaches. First, using an expert-scaling approach, we identified activities and exposures that researchers felt based on prior literature contained a heightened likelihood of potential neurotoxic dust exposures,^4,16,17^ including generalized manual labor, sifting through the debris, working in enclosed and dusty areas, and exposure to chemical and other types of fumes and smoke. The presence of any of these activities was thought to amplify exposure effects unless PPE was used; thus, we calculated a unit-weighted sum of these activities, to make an ordinal exposure variable with 5 distinct categories ranging from low (score, 1) to severe (score, 5) exposure. To validate and accentuate our expert-scaling scoring schema for high-exposure categories, we also used a validated artificial neural network protocol^18,19^ to define a novel empirically derived high-exposure algorithm (eAppendix 1 in Supplement 1). Finally, consistent with a prior study,^14^ we split the neural network moderate- and high-exposure categories based on working for at least 15 weeks on or adjacent to Ground Zero to create an ordinal exposure variable with 5 categories ranging from low (score, 1) to severe (score, 5) exposure. We decided to report findings from the expert-scaling method as main results because they were more readily explained.

### Dementia Diagnosis

Incidence of dementia before age 65 years was the primary outcome and was diagnosed for research purposes using standard diagnostic criteria for all-cause dementia blinded to WTC exposure severity.^20^ Dementia was diagnosed algorithmically by the presence of a new-onset cognitive impairment across 2 domains of cognitive functioning (eg, episodic memory and executive function) accompanied by evidence of functional limitation.^20^ We used the medical history to exclude individuals who had competing medical conditions, including brain cancer, schizophrenia, co-occurring or preexisting neurological conditions, and delirium. Cognitive impairment consistent with dementia was defined as a score lower than 2 SDs below the cohort mean at baseline assessment for episodic memory, as measured using 3 trials of a 5-word verbal learning and recall task (cutoff score <10), and executive function, as measured using a summation of animal fluency, digits-span forward and backward, and trail-making tasks (part B) (cutoff score <8) accompanied by evidence of cognitive decline as indicated by a reduction in global cognition since baseline. Cognitive performance was measured regularly by trained research staff using the Montreal Cognitive Assessment with alternative versions used longitudinally to reduce test-retest bias, and global cognitive performance was measured using the standard scoring algorithm.^21^ Functional limitations were considered present when cognitively impaired responders exhibited difficulty in orienting common objects or recognize common shapes, difficulty in temporal, visuospatial, or contemporary orientation, or in managing simple calculations.

### Covariates

Demographic factors included responder age in years, sex, and educational level (high school diploma or less, some college, or a university degree and higher). Black race and Hispanic ethnicity reportedly increase the risk of dementia;^22^ thus, we accounted for race and ethnicity in this study. Participants were asked to indicate racial identity for 16 fixed categories that were recategorized into 4 mutually exclusive categories (Black, Hispanic, White, and other [including Asian, American Indian or Alaska Native, Hawaiian or Pacific Islander, multiracial, or unknown]).

Medical factors included a medical record or self-reported history of hypertension, diabetes, heart disease, major stroke, other neurological conditions as used for exclusion criteria, any lifetime head injury (classified as none, a single mild injury, at least 1 head injury involving a loss of consciousness, at least 1 head injury diagnosed as a concussion, multiple head injuries with loss of consciousness, and a diagnosis of a concussion). While the incidence of COVID-19 was not associated with exposure, we took this opportunity to examine the sensitivity of analyses to the presence of a diagnosis of mild to severe COVID-19 before the advent of vaccinations, or the presence of postacute sequelae of COVID-19,^23^ thereafter on the diagnosis of dementia.

Lifestyle behavioral factors included smoking history, heavy drinking (≥4 drinks per week), or binge drinking (≥3 drinks per day). Since lifestyle changes can result from disinhibition secondary to neurodegenerative conditions, we used lifetime history of alcohol and smoking at the first cognitive assessment.

Genetic vulnerability for dementia was indicated by apolipoprotein E ε4 (ApoE4) allele possession or using a polygenic risk score for Alzheimer disease (AD) that captures genetic liability across single-nucleotide polymorphisms weighted by effect sizes obtained from a genome-wide association study of AD.^24^ Genetic information was only available for a subgroup of responders who consented to genome-wide analysis.

### Missing Data

Missing data in this study emerged predominantly from the refusal to complete cognitive assessments due to fear of diagnosis. To account for this unique type of missing information, we implemented a weighting method first reported in studies of infectious disease that works by leveraging variability in the effectiveness of research staff to engage research participants in the assessment.^25,26^ Sensitivity analyses also examined the utility of imputing missing data for participants with missing data on covariates.

### Statistical Analysis

Sample characteristics at the initial cognitive assessment were described using means and SDs or frequencies and percentages for the whole sample and stratified by exposure severity group. Nonparametric trend tests and Spearman correlation coefficients were used to compare differences in characteristics across levels of exposure severity. We used robust Poisson regression to estimate multivariable-adjusted risk ratios^27^ comparing demographic and clinical information between responders who participated in cognitive testing with responders who never participated, responders who completed only 1 cognitive assessment with responders who completed more than 1 assessment, and responders who consented to genetic testing with responders who did not. All analyses were weighted to match the demographics of the responder population, and all significance testing relied on 2-tailed *P* values (α = .05 was considered statistically significant).

We calculated crude IRs for the whole sample and then again stratified by level of exposure severity. Risk differences were calculated using incidence rates to provide absolute measures of change. Unadjusted and multivariable-adjusted analyses examined correlates of incidence using Cox proportional hazards regression. The timescale used was the time under observation; responders were censored after 5 years, on the date of death, or at age 65 years. Unadjusted and multivariable-adjusted hazard ratios (AHR) with 95% CIs were reported for each level of exposure severity and as a linear composite to examine exposure trends in incidence. Mean risk difference across all categories was used to estimate trends in absolute risk. Cumulative incidence of dementia was recorded using the number of years since the initial cognitive assessment, which was available starting November 1, 2014. Time-varying covariates included the incidence of stroke during observation and infection with or the presence of postacute symptoms of COVID-19.

Supplemental analyses, detailed in eAppendix 2 in Supplement 1, used Cox proportional hazards regression and Fine-Gray competing risks analysis^28^ to examine whether adjusting for a familial history of all-cause or cause-specific dementia, measured in a subset of responders, influenced the association between exposure severity and risk of dementia. We tested the proportional hazards assumption using Schoenfeld residuals. We also assessed whether possession of the ApoE4 allele changed results in a subset of responders for whom genetic information was available. We considered the impact of age-focused inclusion and exclusion criteria by excluding responders older than 60 years at initial cognitive assessment to ensure the potential for 5 years of follow-up and then excluded those aged 45 years or younger at initial cognitive assessment due to a very low risk of dementia. We examined analytic bias by modifying our timescale in all models as a sensitivity analysis to examine whether the choice of temporal metric changed results. Next, because some researchers like alternative measures of effect size alongside HRs, we calculated etiologic fraction. Finally, we used stratified Mantel-Haenszel homogeneity testing to examine whether results were inconsistent when stratifying data by sociodemographic characteristics. Statistical analyses were completed using Stata/MP 17.0 (StataCorp LLC).

## Results

Among 9891 WTC responders residing on Long Island, New York, who participated in monitoring during our time frame, 78.0% were eligible for this study when cognitive assessments began (Figure 1). After applying inclusion and exclusion criteria, our follow-up response rate was 84.2% (N = 5010; median [IQR] age, 53 (48-57) years; 437 [8.7%] female and 4573 [91.3%] male; and 157 [3.1%] Black, 141 [2.8%] Hispanic, 4369 [87.2%] White, and 342 [6.8%] other race and ethnicity. Participants contributed a mean (SD) of 3.73 (0.87) observations per responder over a total of 15 913.0 person-years.

Compared with participating responders, responders who refused cognitive assessments were younger, were more likely to have diabetes, and were less likely to be current smokers or to have some college or a university degree (eTable 1 in Supplement 1). Responders assessed at baseline who refused follow-up assessments were more likely to be untrained responders at baseline. Responders whose genotyping information was unavailable did not differ from genotyped responders across these measures.

### Sample Characteristics

Sampled responders (Table 1) were in their mid-50s at study commencement, and the majority were male. Most responders were placed in mild to moderate exposure groups. Compared with responders who were moderately exposed, responders who were more heavily exposed were older, less likely to be female, had lower educational attainment, were less likely to be Black, were less likely to report supervisorial activities, and were more likely to be current smokers.

**Table 1.** Baseline Characteristics of the Participating Responders

Characteristic	Participants, No. (%)						*P* value^a^
	Whole sample (n = 5010)	Low exposure or no dust or wore PPE (n = 342)	Mild exposure (n = 2805)	Moderate exposure (n = 1450)	High exposure (n = 324)	Severe exposure (n = 89)	
Age, median (IQR), y	53 (48-57)	54 (49-58)	52 (47-56)	53 (48-57)	55 (38-64)	56 (52-60)	.001
Sex							
Female	437 (8.7)	22 (6.4)	312 (11.1)	94 (6.5)	10 (3.0)	0 (0)	<.001
Male	4573 (91.3)	320 (93.6)	2493 (88.9)	1356 (93.5)	314 (3.1)	89 (100)	
Educational attainment							
≤High school diploma (reference)	1112 (22.2)	55 (16.2)	486 (17.3)	400 (27.7)	133 (40.9)	39 (43.1)	NA
Some college	2430 (48.5)	175 (51.3)	1390 (49.48)	685 (47.41)	140 (42.98)	41 (45.38)	<.001
University degree	1467 (29.28)	111 (32.49)	934 (33.2)	360 (24.9)	52 (16.2)	10 (11.6)	<.001
Race and ethnicity^b^							
Black	157 (3.1)	15 (4.3)	91 (3.2)	45 (3.1)	6 (2.0)	1 (0.7)	.04
Hispanic	141 (2.8)	13 (3.8)	82 (2.9)	36 (2.5)	9 (2.8)	1 (1.1)	.15
White (reference)	4369 (87.2)	287 (84.4)	2452 (87.3)	1264 (87.45)	283 (87.2)	83 (92.5)	NA
Other^c^	342 (6.8)	25 (7.5)	185 (6.6)	100 (6.9)	26 (8.1)	5 (5.7)	.78
Worked as a supervisor	809 (16.1)	120 (35.2)	527 (18.7)	116 (8.0)	38 (11.6)	8 (9.2)	<.001
Untrained responder	1103 (22.0)	42 (12.4)	160 (5.7)	619 (42.8)	202 (62.3)	79 (88.6)	<.001
Hypertension	1401 (28.0)	107 (31.4)	763 (27.2)	417 (28.9)	86 (26.4)	28 (31.7)	.38
Diabetes	428 (8.6)	39 (11.4)	220 (7.8)	131 (9.0)	31 (9.4)	8 (9.0)	.48
Heart disease	388 (7.8)	24 (7.0)	207 (7.4)	126 (8.8)	22 (6.6)	9 (10.2)	.20
Non-WTC head injury history							
No history (reference)	3375 (67.4)	219 (64.2)	1952 (69.5)	941 (65.1)	211 (64.8)	53 (58.7)	NA
Mild head injury	403 (8.0)	27 (8.1)	209 (7.4)	126 (8.7)	33 (10.0)	9 (10.1)	.13
Loss of consciousness	176 (3.5)	16 (4.6)	87 (3.1)	60 (4.1)	12 (3.8)	1 (1.1)	<.001
Concussion	412 (8.2)	39 (11.3)	224 (8.0)	111 (7.7)	30 (9.3)	8 (8.6)	.02
Loss of consciousness and concussion	644 (12.8)	40 (11.8)	338 (12.0)	208 (14.4)	39 (12.1)	19 (21.5)	.01
Smoking history							
Never smoker (reference)	421 (8.4)	21 (6.12)	214 (7.6)	127 (8.8)	48 (14.8)	11 (12.4)	NA
Former smoker	3334 (66.6)	228 (67.0)	1940 (69.0)	933 (64.5)	183 (56.4)	50 (56.0)	<.001
Current smoker	1255 (25.1)	91 (26.8)	656 (23.4)	386 (26.7)	94 (28.8)	28 (31.6)	<.001
Heavy drinker	1572 (31.4)	100 (29.5)	838 (29.8)	493 (34.1)	108 (33.1)	12 (13.6)	.004
Binge drinker	426 (8.5)	30 (8.9)	201 (7.2)	140 (9.7)	42 (12.9)	27 (35.7)	<.001
Apolipoprotein E ε4 allele							
Noncarrier (reference)	3453 (68.9)	244 (71.7)	1929 (68.7)	978 (67.7)	229 (70.4)	72 (80.4)	NA
Heterozygous	843 (16.8)	53 (15.6)	476 (16.9)	253 (17.5)	54 (16.6)	7 (7.8)	.82
Homozygous	60 (1.2)	6 (1.8)	32 (1.1)	18 (1.2)	4 (1.2)	0 (0)	.79
No informed consent or blood unavailable	654 (13.0)	37 (11.0)	373 (13.3)	196 (13.6)	38 (11.7)	11 (11.8)	.72

Abbreviations: NA, not applicable; PPE, personal protective equipment; WTC, World Trade Center.

^a^ *P* < .001 for the comparison between more and less exposed groups calculated using nonparametric trend tests.

^b^ Race and ethnicity were self-reported.

^c^ Other race and ethnicity included Asian, American Indian or Alaskan Native, Hawaiian or Pacific Islander, multiracial, or unknown.

### Incidence of Dementia

During 15 913.1 person-years of follow-up, we identified 228 cases of incident dementia before age 65 years (crude IR, 14.47 [95% CI, 12.74-16.50] per 1000 person-years) that was age graded (eFigure 1 in Supplement 1). Comparing the incidence of dementia across the 5 exposure groupings indicated an incremental increase in the incidence of dementia per 100 person-years (low exposure [ie, no dust exposure or responder wore PPE]: IR, 2.95 [95% CI, 1.07-11.18]; mild: IR, 12.16 [95% CI, 10.09-14.79]; moderate: IR, 16.53 [95% CI, 13.30-20.81]; high: IR, 30.09 [95% CI, 21.35-43.79]; and severe: IR, 42.37 [95% CI, 24.86-78.24]) .

**Figure 2.**
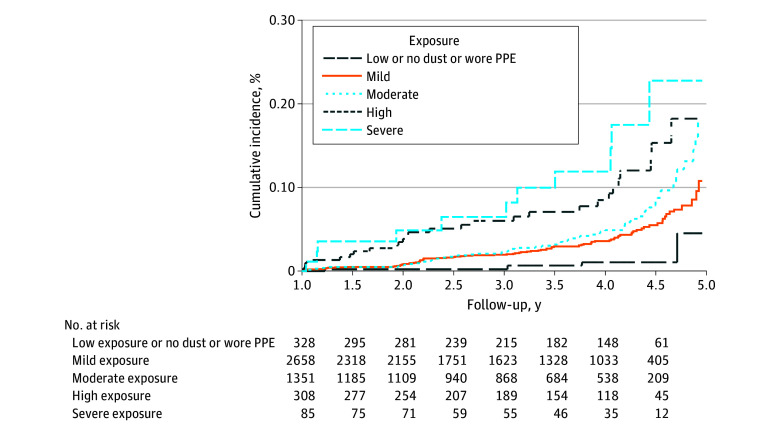
Cumulative Incidence Plot and Accompanying Risk Table for Incidence of Dementia Before Age 65 Years in World Trade Center Responders, Stratified by Exposure Severity Measure PPE denotes consistent usage of personal protective equipment, including body suits and respirators or masks.

Cumulative incidence curves (Figure 2) indicated that responders in the most severely exposed group had elevated hazard of dementia in multivariable-adjusted analyses as compared with responders who reported no dust exposures or wore PPE. Unadjusted differences (Table 2) were statistically significant when examining risk differences (risk difference, 39.42 [95% CI, 11.12-139.70]; *P* < .001) and ratios (AHR, 14.65 [95% CI, 4.29-50.01]; *P* < .001). Multivariable adjusted analyses (Table 2) also indicated that after adjustment for social, demographic, and medical factors, individuals who were more severely exposed had greater risk of dementia. For example, individuals in the highest exposure category had increased hazard of dementia when compared with individuals who had the lowest exposure (AHR, 9.47 [95% CI, 2.70-33.14]; *P* < .001). Multivariable-adjusted trend analyses further indicated that increased WTC exposure was associated with increased hazard of dementia (AHR, 1.42 [95% CI, 1.18-1.71]; *P* < .001; mean risk difference, 9.74 [95% CI, 2.94-32.32] per 1000 person-years; *P* < .001).

**Table 2.** Unadjusted and Multivariable Adjusted Hazard Ratios Examining Incidence of Dementia in World Trade Center Responders, Stratified by Exposure Severity

Exposure group	Incident cases/responders	Person-years of follow-up	IR per 1000 person-years (95% CI)	Risk difference per 1000 person-years (95% CI)	*P* value	Unadjusted HR (95% CI)	*P* value	Demographically adjusted AHR (95% CI)^a^	*P* value	Multivariable adjusted AHR (95% CI)^b^	*P* value
Reference: low exposure or no dust or wore PPE	3/342	1145.0	2.95 (1.07-11.18)	1 [Reference]		1 [Reference]		1 [Reference]		1 [Reference]	
Mild exposure	106/2805	8857.5	12.16 (10.09-14.79)	9.21 (2.92-29.01)	<.001	4.19 (1.51-11.66)	.006	4.45 (1.61-12.32)	.004	4.61 (1.63-13.07)	.004
Moderate exposure	76/1450	4590.2	16.53 (13.30-20.81)	13.58 (4.28-43.04)	<.001	5.56 (1.98-15.68)	.001	5.19 (1.84-14.66)	.002	5.15 (1.78-14.9)	.003
High exposure	31/324	1030.5	30.09 (21.35-43.79)	27.14 (8.3-88.79)	<.001	10.76 (3.59-32.29)	<.001	8.66 (2.91-25.78)	<.001	8.45 (2.80-25.48)	<.001
Severe exposure	12/89	289.8	42.37 (24.86-78.24)	39.42 (11.12-139.7)	<.001	14.65 (4.29-50.01)	<.001	11.05 (3.22-37.96)	<.001	9.47 (2.70-33.14)	<.001
Trend analyses				9.74 (2.94-32.32)	<.001	1.61 (1.36-1.90)	<.001	1.47 (1.22-1.77)	<.001	1.42 (1.18-1.71)	<.001

Abbreviations: AHR, multivariable adjusted hazard ratio; HR, hazard ratio; IR, incidence rate (crude); PPE, personal protective equipment.

^a^ Demographically adjusted hazards accounted for age, sex, race and ethnicity, untrained responder, educational level, and per-recruiter propensity for successful assessment.

^b^ Multivariable adjusted hazard ratios accounted for age, sex, race and ethnicity, untrained responder, educational level, hypertension, diabetes, heart disease, history of head trauma, smoking status, history of heavy or binge drinking, and per-recruiter propensity for successful assessment.

### Neural Networks Validation

Since we were concerned about the potential for insidious exposures, we replicated the work above using a neural network–based approach to score severe exposures (eFigure 2 in Supplement 1 has neural network learning curves). eFigure 3 in Supplement 1 depicts the cumulative incidence of dementia stratified by levels of exposure severity determined using the neural network–based approach. Unadjusted and multivariable-adjusted results (eTable 2 in Supplement 1) were similar in unadjusted (eg, HR, 1.80, [95% CI, 1.53-2.13]; *P* < .001) and multivariable-adjusted (eg, AHR, 1.75, [95% CI, 1.48-2.06]; *P* < .001) analyses.

### Sensitivity Analyses

We examined subsamples with detailed information about a family history of neurodegenerative disease (eFigure 4 in Supplement 1). In sensitivity analyses, accounting for ApoE4 allele possession, population weighting efforts, using subhazard ratios to account for the potentially competing risk of death, changing timescale in analysis or age-based exclusion criteria, or using multiple imputation to account for missing data on confounders did not change the results (eTable 3 in Supplement 1). We reported no statistically significant differences among responders who consistently wore PPE (HR, 0.76 [95% CI, 0.24-2.41]; *P* = .64) or had long exposures without any dust exposure (HR, 0.53 [95% CI, 0.07-4.05]; *P* = .54) (eFigure 5 in Supplement 1). Finally, we tested the homogeneity of results across sociodemographic groups and observed that results did not differ in analyses stratifying for sex, race and ethnicity, training, supervisory status, and educational level.

## Discussion

The intense acute exposure to particulate matter that WTC responders endured in the aftermath of the September 11, 2001, terrorist attacks and the repeated exposures to airborne pollutants and chemicals during the subsequent rescue and recovery operation were associated with increased risk of dementia among the responders who were most severely exposed. Using longitudinal information about 5010 WTC responders 60 years of age or younger at initial assessment, this cohort study found increased risk of dementia associated with working in dusty locations and performing relatively dangerous activities for 15 or more weeks on or adjacent to the pile of debris or pit at Ground Zero. The risk of dementia was lowest for individuals with limited exposure to the WTC sites, responders who worked in low-dust environments, and responders who regularly wore PPE. Our WTC exposure severity score provided the first evidence of an association between WTC exposures and the incidence of dementia. This association remained statistically significant after adjustment for potential confounders, including demographic confounders such as educational level; postexposure psychiatric sequelae, including evidence of re-experiencing stressful memories; the presence of medical diagnoses, including hypertension; and the presence of a history of chronic or severe head injuries.

A growing literature has supported the conclusion that severe exposures to inhaled particulate matter can result in changes in biomarkers.^4^ We expanded prior work to show that responders who consistently wore PPE or never reported dust exposure were associated with relatively low risk of dementia before age 65, while those reporting dangerous exposures were associated with heightened risk of dementia. Results from rodent models suggest that there are changes to olfaction, working memory, visuospatial learning, and behaviors in mice exposed to WTC dust.^29,30^ These results have been replicated in serological studies of humans, which report that cognitively impaired WTC responders have an upregulated macrophagic reaction in pathways indicating neuroimmunologic response,^31^ while neuroimaging studies have found evidence of glial activation and hippocampal inflammation in heavily exposed responders.^32,33^ Seeking potential linkages between neuroinflammation and cognitive outcomes, prior work suggests a potential role for phosphorylated tau and accompanying neurodegeneration.^34^ Further work identifying a mechanism for this process may provide useful treatment targets for WTC responders and other persons with inhaled neurotoxicants.

A central strength of this study is its development of an exposure severity scale that considered the type of work that WTC responders completed, the duration and severity of exposure, and the use of PPE. Efforts by many researchers to recruit an unexposed group have been stymied because most individuals working in response occupations participated in these massive response efforts. Individuals who were excluded from response efforts were often older or in poorer health than persons who responded. This is less concerning because the use of a minimally exposed group, as opposed to an unexposed group, as necessitated when studying this exposure, is most likely to have caused us to underestimate the true association of the WTC exposures with dementia. For example, whereas the IR in the general population is estimated at 1.19 per 1000 person-years among individuals aged 30 to 64 years,^9^ the incidence of dementia in our sample of the least exposed WTC responders still appeared to be somewhat higher (IR, 2.95 per 1000 person-years). Using incidence in the least exposed responders in our sample as the reference group, instead of incidence in the general population, would make our findings more conservative.

We focused on the incidence of all-cause dementia, a rare and heterogeneous disorder with multiple causes that may be associated with markedly shorter lifespan.^35^ Critically, dementia before age 65 years does not have the same risk factors as sporadic dementia observed in older ages. For example, studies often report an association between homozygous ApoE4 possession and risk of dementia before age 65 years that is consistent with that reported in the present study.^36,37^ The sensitivity analyses conducted in the present study replicated previous results showing that polygenic risk scores for AD predicted a higher incidence of dementia in this population,^38^ indicating that neurocognitive deficits of WTC responders may also be sensitive to genetic vulnerability.

### Limitations

This study has limitations. First, the reliance on this cohort of English-speaking WTC responders who enrolled in a congressionally mandated monitoring program implies the potential for differing results when compared with other WTC-affected groups. One replication study of nonresponder WTC survivors, who were not eligible for the present study, recently reported similar associations between dust exposure and cognitive functioning.^15^ However, the extent to which similar patterns would be observed in firefighters, who were more severely exposed but are monitored in an independent program, remains unclear. Second, recall bias can emerge from exposure reports from this large and uncontrolled disaster, and there is no biomarker that measures exposure severity to neurotoxic substances at the WTC. Neuroimaging studies have identified significant differences in neuroinflammation^32,33^ and increased phosphorylated tau 181^34^ in dust-exposed WTC responders who worked for longer durations. Yet the dust that was expelled from the collapse of the Twin Towers was composed of a wide variety of hazardous material, including pulverized glass, lead, polycyclic aromatic hydrocarbons, polychlorinated biphenyls, and dioxins, and may differ from other sources of air pollution. Identifying a biomarker for exposure severity could improve risk measurement here and in the general population. Third, we avoided incorporating measures of closely linked psychiatric disorders, including posttraumatic stress disorder, generalized anxiety disorder, and major depression. In developmental work leading up to this study, we noted that symptoms of depression and anxiety are often colinear and that posttraumatic stress disorder and depressive symptoms are increased in individuals with mild cognitive impairment.^39^ Fourth, we excluded individuals with diagnoses of dementia at first cognitive assessment because we could not determine their timing of disease. This ensured that dementia onset occurred after exposure and confounder identification but also likely excludes the most severe cases. Finally, we did not determine the etiology or subtype of dementia in this study. One reason we focused on dementia before age 65 years is that it reduces the potential influence of AD and cerebrovascular diseases predominantly affecting older adults. Future work should improve the differential diagnosis of exposure-related dementia.

## Conclusions

The WTC disaster required an immediate and prolonged response to an unexpected tragic event in dangerous conditions, and most response personnel completed these tasks as part of their everyday duties. This study builds on prior work suggesting that dust and debris from the WTC collapse contained neurotoxins. This cohort study found that among WTC responders who survived the events of September 11, 2001, to first participate in the WTC response efforts and then to enroll in a longitudinal follow-up study of cognition from 2014 through 2022, detailed reports of their types and durations of exposures, response activities, and use of PPE were associated with incidence of dementia before age 65 years. When compared with the lowest reported exposure levels or use of PPE, a higher level of exposure to more dust and debris was significantly associated with an elevated risk of dementia before age 65 years. There is a critical need to protect persons who help in rescue and recovery operations after an unexpected industrial accident. These results imply that these exposures were dangerous and support the view that the use of PPE might have prevented the onset of dementia before age 65 years among exposed responders. Future research is warranted to determine cerebral biomarkers for individuals with exposure-related dementia.

## Notes

Supplement 1.
eMethods.
**eFigure 1.** Age-specific incidence rates stratified by five-year age group at baseline for responders in this analysis **eTable 1.** Selection bias analyses showing association between all covariates and the risk of missing cognitive assessment at baseline, a second follow-up assessment, and genetic information **eAppendix 1.** Validation effort using a secondary neural network risk scoring metric **eFigure 2.** Learning curves indicating the artificial neural network’s ability to rely on exposure information to identify individuals with dementia in training and testing **eFigure 3.** Nelson-Aalen plot and accompanying risk table showing cumulative hazards of dementia before age 65 years in World Trade Center responders, stratified by probable exposure severity as determined using a combination of human and computer-driven scoring methods for probable exposures while working on-site in the aftermath of the terrorist attacks of 9/11/2001 **eTable 2.** Unadjusted and multi-variable adjusted hazard ratios examining incidence of dementia in World Trade Center responders, stratified by exposure severity **eAppendix 2.** Supplemental analyses **eFigure 4.** Bar graph showing hazards ratios for early-onset dementia by level of exposure after adjusting for age and family history of amyotrophic lateral sclerosis, Huntington’s disease, spinocerebral ataxia, or multisystem atrophy **eTable 3.** Sensitivity analyses showing trend-level associations between qualitative exposure metric and hazards of early-onset dementia when adjusting for additional covariates that were only available in a subset of individuals **eFigure 5.** Nelson-Aalen plot and accompanying risk table showing cumulative hazards of dementia before age 65 years in minimally exposed World Trade Center responders, stratified by whether a person report no significant dust exposure or usually wore personal protective equipment as compared to those in low-risk activities while working on-site in the aftermath of the terrorist attacks of 9/11/2001

Supplement 2.
Data Sharing Statement
