# Supplementary material for: Incidence of Dementia Before Age 65 Years Among World Trade Center Attack Responders
Source: JAMA Netw Open. 2024 Jun 12;7(6):e2416504. doi: 10.1001/jamanetworkopen.2024.16504 (PMC11170296; doi:10.1001/jamanetworkopen.2024.16504)
Supplement: Supplement 1. — eMethods. eFigure 1. Age-specific incidence rates stratified by five-year age group at baseline for responders in this analysis eTable 1. Selection bias analyses showing association between all covariates and the risk of missing cognitive assessment at baseline, a second follow-up assessment, and genetic information eAppendix 1. Validation effort using a secondary neural network risk scoring metric eFigure 2. Learning curves indicating the artificial neural network’s ability to rely on exposure information to identify individuals with dementia in training and testing eFigure 3. Nelson-Aalen plot and accompanying risk table showing cumulative hazards of dementia before age 65 years in World Trade Center responders, stratified by probable exposure severity as determined using a combination of human and computer-driven scoring methods for probable exposures while working on-site in the aftermath of the terrorist attacks of 9/11/2001 eTable 2. Unadjusted and multi-variable adjusted hazard ratios examining incidence of dementia in World Trade Center responders, stratified by exposure severity eAppendix 2. Supplemental analyses eFigure 4. Bar graph showing hazards ratios for early-onset dementia by level of exposure after adjusting for age and family history of amyotrophic lateral sclerosis, Huntington’s disease, spinocerebral ataxia, or multisystem atrophy eTable 3. Sensitivity analyses showing trend-level associations between qualitative exposure metric and hazards of early-onset dementia when adjusting for additional covariates that were only available in a subset of individuals eFigure 5. Nelson-Aalen plot and accompanying risk table showing cumulative hazards of dementia before age 65 years in minimally exposed World Trade Center responders, stratified by whether a person report no significant dust exposure or usually wore personal protective equipment as compared to those in low-risk activities while working on-site in the aftermath of the terrorist attacks of 9/11/ [file jamanetwopen-e2416504-s001.pdf]

Supplementary Online Content

Clouston SAP, Mann FD, Meliker J, et al. Incidence of dementia before age 65 years among World Trade Center attack responders. *JAMA Netw Open*. 2024;7(6):e2416504.
doi:10.1001/jamanetworkopen.2024.16504

eMethods.
eFigure 1. Age-specific incidence rates stratified by five-year age group at baseline for responders in this analysis
eTable 1. Selection bias analyses showing association between all covariates and the risk of missing cognitive assessment at baseline, a second follow-up assessment, and genetic information
eAppendix 1. Validation effort using a secondary neural network risk scoring metric
eFigure 2. Learning curves indicating the artificial neural network's ability to rely on exposure information to identify individuals with dementia in training and testing
eFigure 3. Nelson-Aalen plot and accompanying risk table showing cumulative hazards of dementia before age 65 years in World Trade Center responders, stratified by probable exposure severity as determined using a combination of human and computer-driven scoring methods for probable exposures while working on-site in the aftermath of the terrorist attacks of 9/11/2001
eTable 2. Unadjusted and multi-variable adjusted hazard ratios examining incidence of dementia in World Trade Center responders, stratified by exposure severity
eAppendix 2. Supplemental analyses
eFigure 4. Bar graph showing hazards ratios for early-onset dementia by level of exposure after adjusting for age and family history of amyotrophic lateral sclerosis, Huntington's disease, spinocerebral ataxia, or multisystem atrophy
eTable 3. Sensitivity analyses showing trend-level associations between qualitative exposure metric and hazards of early-onset dementia when adjusting for additional covariates that were only available in a subset of individuals
eFigure 5. Nelson-Aalen plot and accompanying risk table showing cumulative hazards of dementia before age 65 years in minimally exposed World Trade Center responders, stratified by whether a person report no significant dust exposure or usually wore personal protective equipment as compared to those in low-risk activities while working on-site in the aftermath of the terrorist attacks of 9/11/2001
eReferences.

This supplementary material has been provided by the authors to give readers additional information about their work.

eMethods.

Power Analysis

We expected a twofold increase in the incidence of dementia in responders aged ≤65. Based on expectations, a power analysis suggested that with an expected observational period of up to five years, we would need approximately 55 new cases of dementia to detect an unadjusted hazard ratio of 2.7. A sample size of 5,000 responders was considered sufficient to provide power to detect a 2.7-fold difference after adjusting for covariates and attrition, and a timeline for data collection was generated that resulted in the present analysis of 5,010 responders over five years whose follow-up information was recorded by 12/31/2022.

Missing Data

Most missing data in this study emerge from people refusing to complete the cognitive assessments, which may be due to the risk of being diagnosed with a potentially stigmatizing diagnosis. To account for this unique type of missing information, we implemented a weighting method first reported in studies of infectious disease that works by leveraging variability in successful research engagement [1,2]. This method then suggests relying on Probit regression to estimate the probability of cognitive assessment completion based on the recruiter success rate and incorporate the probability of study recruitment in covariate adjustment.

From covariates, many of which are asked multiple times over the course of a longitudinal study, we were missing information for 16 potential participants. Sensitivity analyses to examine the importance of including or excluding these individuals found that excluding them did little to change either the incidence rate and did not change the risk associated with

© 2024 Clouston SAP et al. *JAMA Network Open*.

eFigure 1. Age-specific incidence rates stratified by five-year age group at baseline for responders in this analysis

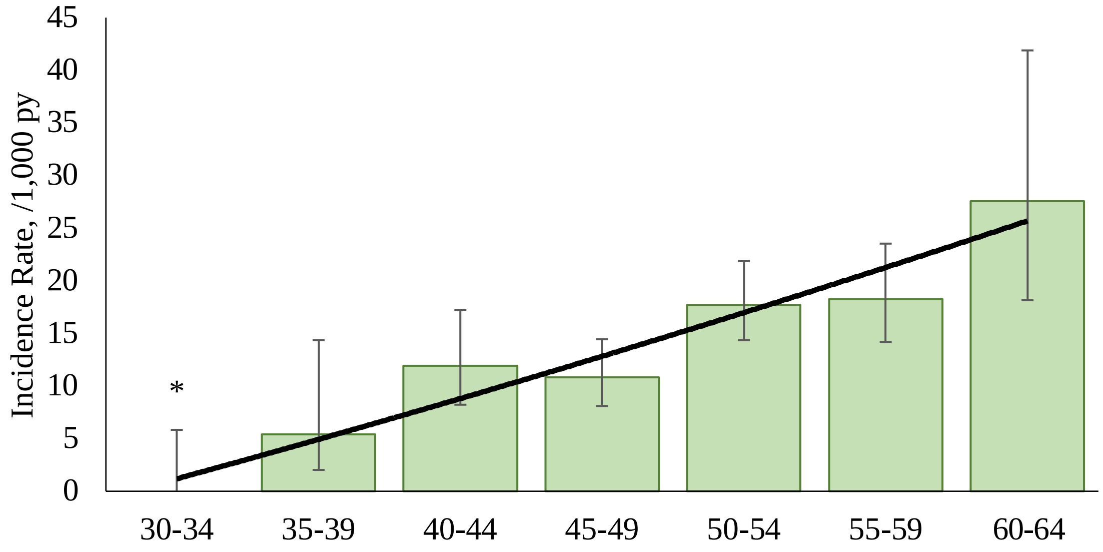

Note: py: person-years, Incidence rates (IR) shown here are unadjusted for covariates. Age is measured at the responder's initial assessment. *Error bars for the 30-34 age group were estimated using backward projection because risk is low, and no early onset dementias were recorded in this relatively small group.

© 2024 Clouston SAP et al. *JAMA Network Open*.

eTable 1. *Selection bias analyses showing association between all covariates and the risk of missing cognitive assessment at baseline, a second follow-up assessment, and genetic information*

	Missing Cognitive Assessment at Baseline			Missing Follow-Up Information			Missing Genetic Information		
	aRR	95% C.I.	P-Value	aRR	95% C.I.	P-Value	aRR	95% C.I.	P-Value
Exposure Severity	1.075	0.977-1.184	0.14	0.978	0.96-0.997	0.02	1.006	0.992-1.02	0.42
Age – yr	0.988	0.975-1.002	0.09	0.993	0.99-0.995	<0.001	0.997	0.995-0.999	0.004
Female sex	0.946	0.699-1.28	0.72	0.963	0.911-1.018	0.18	0.988	0.949-1.029	0.56
Educational Attainment									
High School Diploma or less	1.000			1.000					
Some College	0.907	0.738-1.114	0.35	1.030	0.987-1.075	0.17	0.998	0.967-1.03	0.91
University Degree	0.851	0.673-1.077	0.18	1.013	0.967-1.062	0.59	1.005	0.971-1.04	0.78
Race/Ethnicity									
White	1.000			1.000			1.000		
Black	1.146	0.8-1.643	0.46	0.908	0.838-0.984	0.02	0.979	0.921-1.04	0.49
Hispanic	0.672	0.414-1.092	0.11	0.633	0.56-0.716	<0.001	0.794	0.708-0.89	<0.001
Other	1.581	1.203-2.079	0.001	1.033	0.973-1.096	0.29	1.083	1.048-1.12	<0.001
Worked as a Supervisor	1.005	0.787-1.283	0.97	1.006	0.962-1.051	0.80	0.991	0.957-1.027	0.63
Untrained Responder	1.030	0.833-1.274	0.79	0.984	0.942-1.027	0.45	0.958	0.925-0.992	0.02
Intrusive Stressful Memories – Sx	1.166	1.073-1.266	<0.001	0.977	0.958-0.996	0.02	1.010	0.995-1.024	0.20
Hypertension	1.154	0.956-1.392	0.14	0.929	0.894-0.966	<0.001	0.963	0.935-0.992	0.01
Diabetes	1.105	0.833-1.466	0.49	0.962	0.902-1.027	0.25	0.968	0.919-1.02	0.22
Heart Disease	0.872	0.62-1.227	0.43	1.077	1.017-1.142	0.01	1.045	1.002-1.089	0.04
Non-WTC Head Injury History									
No History	1.000			1.000			1.000		
Mild head injury	0.819	0.591-1.135	0.23	1.033	0.976-1.092	0.27	1.053	1.012-1.096	0.01
Loss of consciousness	0.714	0.425-1.201	0.21	1.182	1.101-1.268	<0.001	1.126	1.078-1.176	<0.001
Concussion	1.169	0.88-1.554	0.28	1.077	1.021-1.137	0.006	1.032	0.99-1.075	0.14
Loss of consciousness and concussion	1.116	0.879-1.417	0.37	1.081	1.033-1.131	0.001	1.095	1.064-1.127	<0.001
Smoking History									
Never Smoker	1.000			1.000					
Former Smoker	1.026	0.759-1.387	0.87	1.152	1.074-1.235	<0.001	1.043	0.99-1.099	0.12
Current Smoker	1.031	0.748-1.422	0.85	1.196	1.112-1.286	<0.001	1.058	1.002-1.118	0.04
Heavy Drinker	1.081	0.899-1.301	0.41	0.995	0.961-1.031	0.79	0.982	0.957-1.009	0.19
Binge Drinker	0.869	0.634-1.191	0.38	0.998	0.94-1.059	0.94	0.979	0.934-1.025	0.37

Note: a*RRs represent multivariable-adjusted risk ratios. Results are from a Poisson regression with a robust standard error.*

© 2024 Clouston SAP et al. *JAMA Network Open*.

eAppendix 1. *Validation effort using a secondary neural network risk scoring metric*

We were concerned by the potential that we would misattribute the most severe risks because they may either be relatively innocuous for responders or might either have unanticipated, nonlinear effects or may have effects that were subject to moderation. We therefore not only relied on results from our expert-rated scores shown above to assess severe risk, but also examined a computer-assisted approach that used a neural network to examine prevalent cases of disease, which were excluded from this study. The neural network is a backpropagation network with two hidden layers each consisting of ten synapses each, alongside 68 input exposure features and a single risk score ranging from one deemed to have high risk, to zero deemed not to be at high risk. To train the Neural network, at each epoch we split the sample into random samples made up of 80% of the sample (for training) and 20% of the sample (for testing conditions) to retain the most power possible for training purposes. Within each epoch, the neural network was presented with 1,000 training occasions and data were randomly sorted each time between occasions. The area under the receiver-operating curve was reported for the training sample, and the score was estimated for the remaining 20% of the sample and are reported in Supplemental Figure 3 as learning curves. When accuracy in the replication sample was reliably close (⎬AUC≤0.10) to the training sample, the algorithm was halted, and the neural network was stored.

These analyses amplified trend results among those most severely exposed in unadjusted and in multivariable-adjusted analyses such that responders with the severe but shorter exposures had elevated hazards of dementia before age 65 years, while those with severe exposures lasting ≥15 weeks had drastically elevated hazards of dementia.

© 2024 Clouston SAP et al. *JAMA Network Open*.

eFigure 2. Learning curves indicating the artificial neural network's ability to rely on exposure information to identify individuals with dementia in training and testing. Training is the black dashed line and 95% confidence interval; five-epoch moving average provided in red dashed lines; testing is solid black line with 95% confidence interval [gray lines]; five-epoch moving average provided in red solid line.

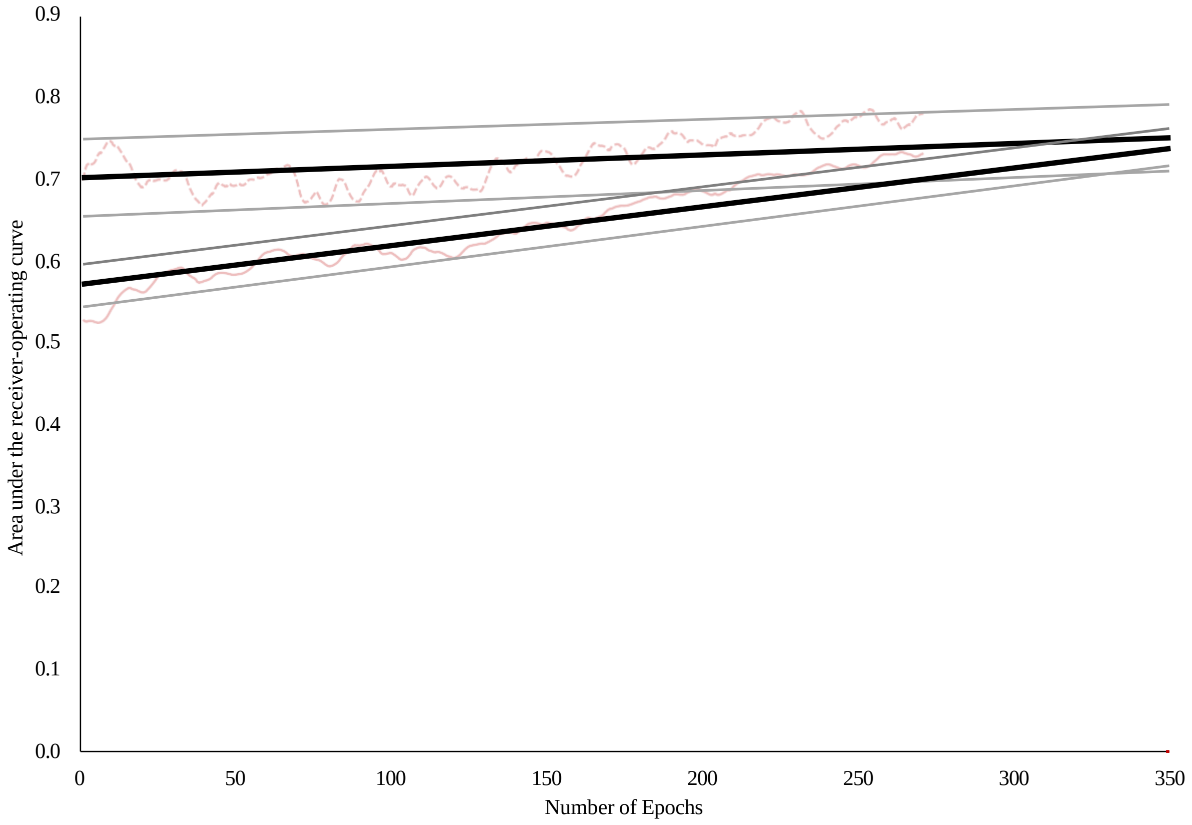

© 2024 Clouston SAP et al. *JAMA Network Open*.

eFigure 3. Nelson-Aalen plot and accompanying risk table showing cumulative hazards of dementia before age 65 years in World Trade Center responders, stratified by probable exposure severity as determined using a combination of human and computer-driven scoring methods for probable exposures while working on-site in the aftermath of the terrorist attacks of 9/11/2001.

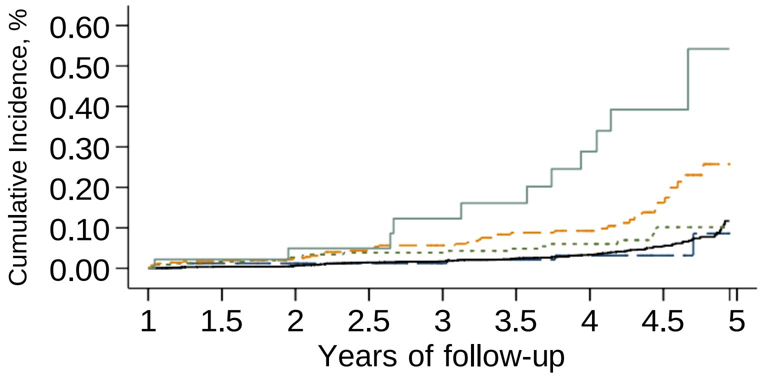

Number at risk								
Low Exposure/No Dust or Wore PPE	179	159	151	128	113	103	84	33
Moderate Exposure for <15 wks	3930	3437	3197	2637	2431	1968	1532	606
Moderate Exposure for ≥15 wks	331	288	271	233	221	176	136	51
High Exposure for <15 wks	432	385	363	296	273	218	174	67
High Exposure for ≥15 wks	45	41	37	25	24	21	17	4

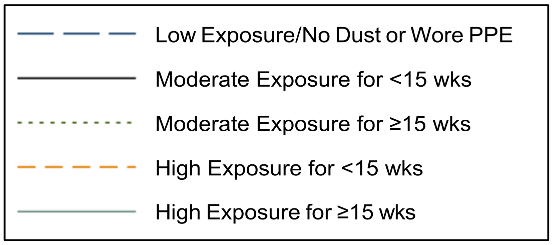

Note: Vertical black tick marks show censoring occasions. Low-risk activities and those who were not exposed to dust or who wore full personal protection equipment always when working on-site shown in dashed navy lines. Those who completed moderately risky activities for shorter periods of time (≤15 weeks) shown in black. Those who completed moderately risky activities for lengthy periods of time (>15 weeks) shown in dashed green line. Those with severe activities for shorter periods of time (≤15 weeks) shown in yellow dashed lines. Those with severe exposure activities for lengthy periods of time (>15 weeks) shown in solid teal. Number at risk at each time-point shown in the risk table.

© 2024 Clouston SAP et al. *JAMA Network Open*.

eTable 2. Unadjusted and multi-variable adjusted hazard ratios examining incidence of dementia in World Trade Center responders, stratified by exposure severity							
**Exposure Group**	**Incident Cases / Responders**	**Person-years of follow-up**	**Incidence Rate /1,000 p-y [95% C.I.]**	**Risk Difference /1,000 p-y [95% C.I.]**	**Unadjusted Hazard Ratio [95% C.I.] P-Value**	**Demographics Adjusted Hazard Ratio [95% C.I.] P-Value**	**Multivariable Adjusted Hazard Ratio [95% C.I.] P-Value**
Low Risk/No Dust or Wore PPE	4/344	595.1	7.32 [3.02-22.56]	0.00	1.00	1.00	1.00
Moderate Exposure for <15 wks	145/722	12,692.4	11.45 [9.76-13.52]	4.13 [2.59, 6.60] P<0.001	1.58 [0.63-3.92] P=0.327	1.58 [0.64-3.87] P=0.115	1.60 [0.65-3.98] P=0.308
Moderate Exposure for ≥15 wks	20/2296	1,088.3	17.40 [11.29-28.29]	10.09 [5.97, 17.05] P<0.001	2.39 [0.85-6.73] P=0.099	2.19 [0.79-6.02] P=0.029	2.15 [0.77-5.96] P=0.144
Severe Exposure for <15 wks	46/1572	1,396.8	32.79 [24.94-44.02]	25.48 [9.17, 70.78] P<0.001	4.55 [1.74-11.89] P=0.002	4.64 [1.80-11.99] P<0.001	4.63 [1.77-12.11] P=0.002
Severe Risk for >15 wks	13/353	140.5	94.05 [57.37-164.77]	86.73 [28.28, 266.01] P<0.001	13.60 [4.58-40.41] P<0.001	12.01 [4.10-35.22] P<0.001	10.84 [3.63-32.4] P<0.001
Summary and Dose-Response Analyses			14.31 [12.6-16.32]	21.68 [7.91-61.82]	1.80 [1.53-2.13] P<0.001	1.78 [1.51-2.11] P<0.001	1.75 [1.48-2.06] P<0.001

***Note**: Multi-variable hazard ratios adjusted for age, gender, race/ethnicity, hypertension, diabetes, history of stroke, history of heart attack, history of head trauma, smoking status, history of heavy or binge drinking, and the propensity for the recruiter to fail to recruit participants. PPE: Personal protective equipment; py: person-years; wks: weeks on or adjacent to pile; IR: Crude Incidence Rate; HR: unadjusted hazards ratio; aHR: multivariable adjusted hazards ratio; 95% C.I.: 95% confidence interval.

© 2024 Clouston SAP et al. *JAMA Network Open*.

eAppendix 2. *Supplemental analyses*

While not available for everyone because of a delay in its inclusion in the study, a detailed family history was collected indicating the presence of any cognitively impairing disease including all-cause and cause-specific dementia was reported by a subset of WTC responders after its addition to the research program. A family history of all-cause dementia was not associated with exposure (ξ^2 = 0.245, P=0.993) and therefore does not satisfy a central criterion for confounding. A family history of all-cause dementia was also not associated with a higher incidence of dementia in this study (HR = 0.90 [0.65-1.25], P = 0.528). When entered in a multivariable-adjusted statistical model these variables did not explain the trend attributed to higher exposure as noted by small differences between models, so we did not integrate these analyses into the reported study.

© 2024 Clouston SAP et al. *JAMA Network Open*.

eFigure 4. Bar graph showing hazards ratios for early-onset dementia by level of exposure after adjusting for age and family history of amyotrophic lateral sclerosis, Huntington's disease, spinocerebral ataxia, or multisystem atrophy

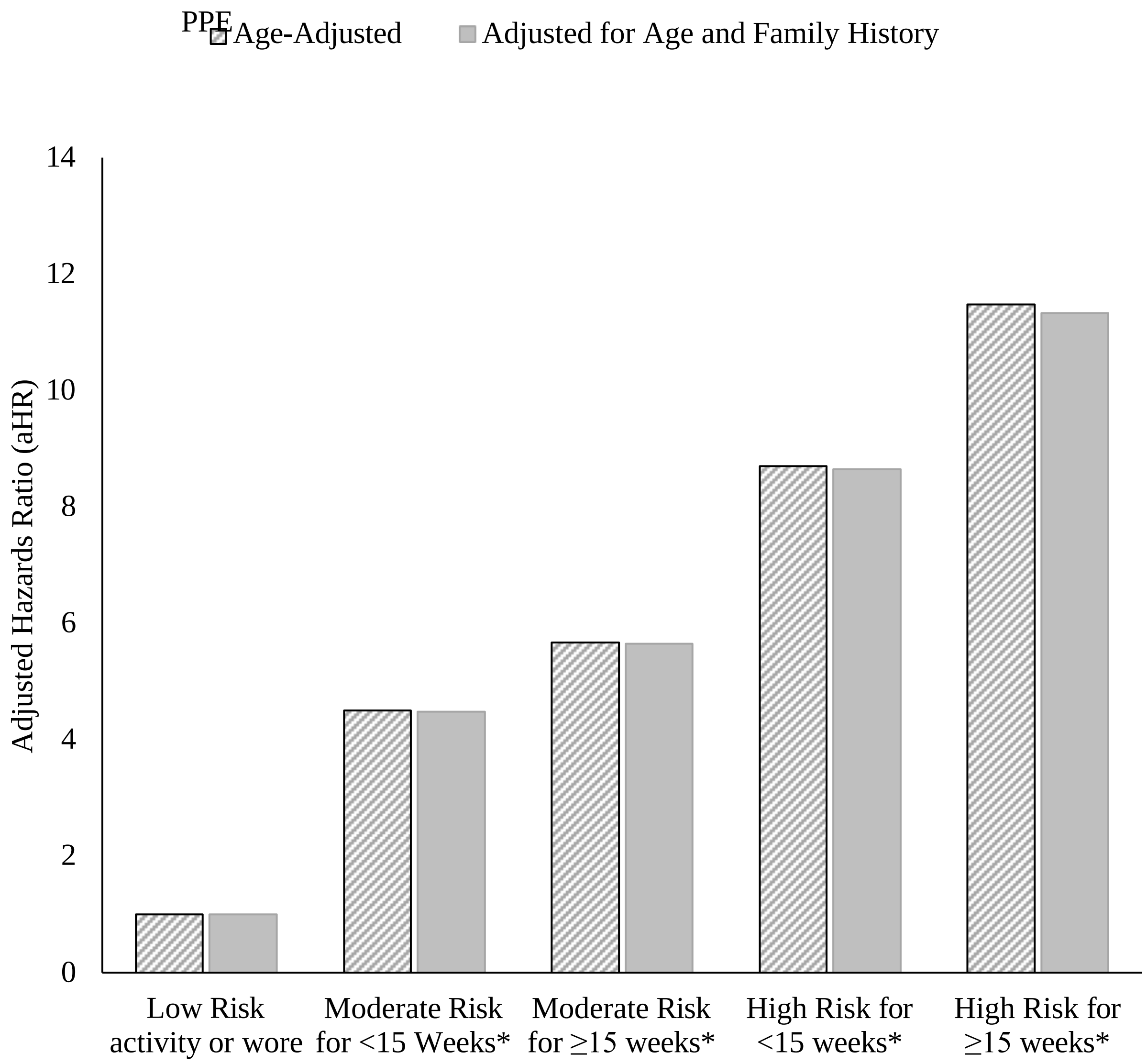

© 2024 Clouston SAP et al. *JAMA Network Open*.

The sensitivity of the presented models to analytic decisions was a question of intense interest to research staff. We began by adjusting simply for markers of familial vulnerability to the disease by adjusting for, in subset analyses, family history and possession of the apolipoprotein-e4 allele. Next, we examined whether stratification by age of onset changed results. We considered the necessity of adjusting for COVID diagnostic status as a time-varying covariate; while COVID is ever present, there is no evidence that infection or infection severity is associated with WTC exposures nor that COVID causes dementia in young healthy people. Nevertheless, we examined the necessity of considering COVID because of its outsized influence on daily life during this period. Next, we considered the potential biasing influence of mortality in this cohort by using the Fine-Gray method to calculate sub-hazard ratios (SHR) [3], while accounting for mortality timing among 25 people who died prior to diagnosis and provided, for comparison, SHR not adjusting for death. While the SHR is not as high or as statistically significant, in general, it is similar when we adjusted for mortality as compared to when using the same method but when all deaths were censored but were not registered as competing risks. Finally, we considered the importance of using different time scales. Results supported reliance on the models that were already being reported.

© 2024 Clouston SAP et al. *JAMA Network Open*.

eTable 3. Sensitivity analyses showing trend-level associations between qualitative exposure metric and hazards of early-onset dementia when adjusting for additional covariates that were only available in a subset of individuals

Model Adjustment	aHR	95% C.I.	P
Multiple imputation for missing data	1.49	[1.28–1.75]	<0.001
Genetic and Familial Vulnerability			
Adjusting for Apolipoprotein-e4 Status	1.47	[1.20–1.80]	<0.001
Adjusting for Family History of All-Cause Dementia	1.42	[1.14–1.78]	0.002
COVID-19 Diagnosis			
COVID-19 diagnosis as Time-Varying Covariate	1.47	[1.22–1.78]	<0.001
Competing Risks Analysis			
Sub-hazard Ratio not adjusted for competing mortality*	1.28	[1.06–1.54]	0.010
Sub-hazard Ratio adjusting for competing mortality*	1.28	[1.06–1.54]	0.010
Sample Limitations			
≤60 Years Old at First Cognitive Assessment	1.44	[1.18–1.75]	<0.001
≥45 Years Old at First Cognitive Assessment	1.42	[1.16–1.73]	0.001
Recall Bias Assessment			
Exposure assessed before 9/11/2011	1.60	[1.22–2.11]	<0.001
Exposure assessed after 9/11/2011	1.67	[1.35–2.07]	<0.001
Time Scale			
Years since 9/11/2001	1.50	[1.21–1.84]	<0.001
Age in years	1.50	[1.25–1.81]	<0.001

Note: *Reports a demographically adjusted SHR (Subhazard Ratio) using the Fine-Gray method. COVID-19: Coronavirus Disease of 2019, aHR: demographically adjusted hazards ratio; 95% CI: 95% confidence interval; P: P-value.

© 2024 Clouston SAP et al. *JAMA Network Open*.

eFigure 5. Nelson-Aalen plot and accompanying risk table showing cumulative hazards of dementia before age 65 years in minimally exposed World Trade Center responders, stratified by whether a person report no significant dust exposure or usually wore personal protective equipment as compared to those in low-risk activities while working on-site in the aftermath of the terrorist attacks of 9/11/2001

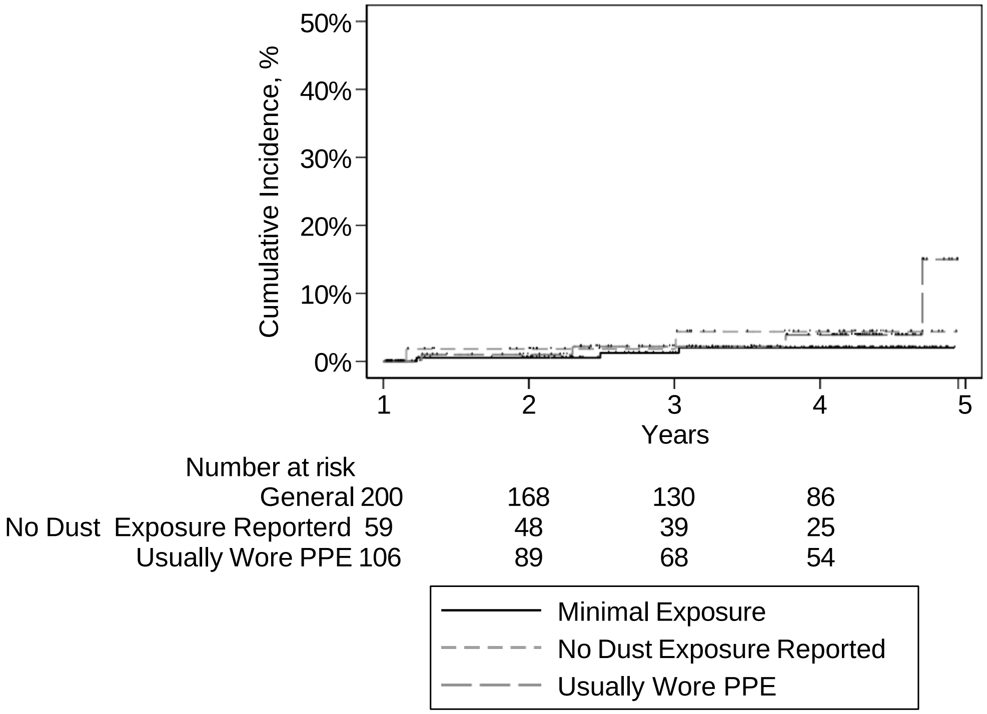

© 2024 Clouston SAP et al. *JAMA Network Open*.

eReferences

1. Benjamini Y, Hochberg Y. Controlling the false discovery rate: a practical and powerful approach to multiple testing. *Journal of the Royal statistical society: sreies B (Methodological)* 1995; **57**(1): 289-300.
2. Arpino B, De Cao E, Peracchi F. Using panel data for partial identification of human immunodeficiency virus prevalence when infection status is missing not at random. *Journal of the Royal Statistical Society: Series A (Statistics in Society)* 2014; **177**(3): 587-606.
3. Fine JP, Gray RJ. A proportional hazards model for the subdistribution of a competing risk. *Journal of the American statistical association* 1999; **94**(446): 496-509.

© 2024 Clouston SAP et al. *JAMA Network Open*.
