# Supplementary material for: Incidence of Dementia Before Age 65 Years Among World Trade Center Attack Responders
Source: JAMA Netw Open. 2024 Jun 12;7(6):e2416504. doi: 10.1001/jamanetworkopen.2024.16504 (PMC11170296; doi:10.1001/jamanetworkopen.2024.16504)
Supplement: Supplement 2. — Data Sharing Statement [file jamanetwopen-e2416504-s002.pdf]

Data Sharing Statement

Data
Data available: Yes
Data types: Deidentified participant data
How to access data: sean.clouston@stonybrookmedicine.edu
When available: With publication

Supporting Documents
Document types: None

Additional Information
Who can access the data: Anyone whose proposed use of the data has been received and approved.
Types of analyses: Any purpose of interest to the WTC responder population.
Mechanisms of data availability: After completion of a signed data access agreement.
Any additional restrictions: No specific dates can be released publicly.
